# Morphology, Not Motion: Benchmarking Vision-Language Models on Multi-Sign Lung Ultrasound Interpretation

**DOI:** 10.64898/2026.07.23.26358829

**Published:** 2026-07-27

**Authors:** Jaeyeon Lee, Athanasios Papastathopoulos-Katsaros, Ilia Buralkin, Zahid Shaik, Brandon Lee, Stephanie Ka-Wing Leung, Michael Alavi, Benjamin Silva, Benjamin Choi, Zhandong Liu, Hyun-Hwan Jeong

## Abstract

**Background:** Point-of-care lung ultrasound (LUS) interpretation requires specialized training. Vision-language models (VLMs) may automate multi-sign assessment but lack systematic clinical benchmarking. We benchmarked three LUS tasks on 125 evaluable cases drawn from 150 point- of-care ultrasound (POCUS) Atlas clips to establish baseline capabilities and isolate clinically relevant failure modes for VLM-based LUS interpretation. **Methods:** We evaluated a normalized multi-model benchmark on three tasks: pleural sliding detection (T1), lung-rocket (B-line) and consolidation classification (T2), and Posterolateral Alveolar and/or Pleural Syndrome (PLAPS; comprising posterolateral consolidation and pleural effusion) assessment (T3). Inputs were 10 uniformly sampled frames for all tasks, plus synthetic M-mode images for sliding. **Results:** We report four findings. First, M-mode improved T1 sliding over static frames for some models, but performance remained modest with wide uncertainty. Second, T2 pathology, identifiable from single frames, was discriminated most clearly above chance on a balanced cohort, with strong lung-rocket and anterior-consolidation F1 achieved by open-weight rather than closed-weight Claude models, although a significant between-model difference held only for lung rockets. Third, T3 PLAPS F1 was high for most models but reflected high positive-class prevalence rather than strong discrimination. Fourth, inter-model agreement was near chance (task-averaged Cohen’s *κ* ≈ 0.02-0.29) and well below each model’s self-consistency, indicating distinct, non-redundant error patterns. **Conclusions:** Static-frame VLMs produced discriminative labels for B-lines and anterior consolidation but remained unreliable for motion-dependent signs (pleural sliding) and prevalence-inflated outcomes (PLAPS). Synthetic M-mode partially recovered temporal information but did not achieve clinically sufficient sliding accuracy. These results support morphology-assisted use for B-lines and consolidation; motion-dependent signs and balanced endpoints require larger validation cohorts before clinical deployment.

## Full Text Availability

The license terms selected by the author(s) for this preprint version do not permit archiving in PMC. The full text is available from the preprint server.

